# Comparative Analysis of Structurally Diverse PFAS-Induced Injury in Vascular Endothelial Cells and Characterization of Necroptosis-Related Cell Death Signaling

**DOI:** 10.3390/toxics14060510

**Published:** 2026-06-11

**Authors:** Sayori Ichijo, Toshiyuki Kaji, Tomoya Fujie

**Affiliations:** Laboratory of Environmental Health, Faculty of Pharmaceutical Sciences, Tokyo University of Science, 6-3-1 Niijuku, Katsushika-ku, Tokyo 125-8585, Japan

**Keywords:** PFAS, PFOS, PFDA, vascular endothelial cell, cellular injury, structure–activity relationship, necroptosis

## Abstract

Perfluoroalkyl and polyfluoroalkyl substances (PFAS) are persistent environmental contaminants associated with cardiovascular diseases; however, the mechanisms underlying PFAS-induced vascular endothelial injury remain incompletely understood. In this study, we systematically evaluated the effects of 15 PFAS on endothelial morphology and cell viability with different carbon-chain lengths and functional groups in cultured bovine aortic endothelial cells. Morphological observations and MTT assays revealed that perfluorononanoic acid, perfluorodecanoic acid (PFDA), and perfluorooctane sulfonate (PFOS) markedly reduced cell viability, with estimated concentrations producing a 50% reduction in viability of 60.9, 34.7, and 87.3 µM, respectively, whereas the other tested PFAS did not reduce viability by 50% at concentrations up to 100 µM in bovine aortic endothelial cells. Among the perfluoroalkyl carboxylic acids, the reduction in cell viability increased with increasing carbon-chain length. Among perfluoroalkyl sulfonates, PFOS caused the greatest reduction in cell viability, whereas perfluorodecanesulfonate did not induce clear endothelial damage. Comparative analyses across multiple cell types showed that PFDA reduced cell viability broadly across all cell types examined, whereas PFOS caused a greater reduction in cell viability in bovine-derived cell types examined than in human- or porcine-derived cell types examined. Since PFDA and PFOS were the most cytotoxic compounds among perfluoroalkyl carboxylic acids and perfluoroalkyl sulfonates, respectively, in bovine aortic endothelial cells, they were selected to compare cell death signaling. In both PFOS- and PFDA-treated cells, the selected apoptosis- and pyroptosis-related markers were not altered under the tested conditions. PFDA was associated with increases in phosphorylated RIP3 and phosphorylated MLKL, whereas PFOS increased MLKL expression without detectable RIP3 activation. Inhibition experiments further suggested that necroptosis-related signaling contributes, in part, to PFOS- and PFDA-induced endothelial injury in vascular endothelial cells. These findings suggest that PFAS-induced vascular endothelial injury depends on molecular structure and cell type, and may involve distinct necroptosis-related signaling patterns. However, it should be noted that the PFAS concentrations used in this study were higher than those typically detected in environmental and human exposure settings.

## 1. Introduction

Perfluoroalkyl and polyfluoroalkyl substances (PFAS) are a broad class of synthetic chemicals defined by the presence of at least one fully fluorinated methyl (–CF_3_) or methylene (–CF_2_–) group in their molecular structure [1]. Thousands of PFAS have been developed and are widely used in industrial and consumer products owing to the exceptional chemical stability conferred by strong carbon–fluorine bonds [2]. Among them, perfluorooctanoic acid (PFOA) and perfluorooctane sulfonate (PFOS) are the most typical PFAS and are widely used in firefighting foams, food packaging materials, and fluoropolymer-coated cookware [3]. Biomonitoring data from the U.S. National Health and Nutrition Examination Survey (NHANES) consistently detected PFAS in serum across all age groups [4,5], underscoring global concern regarding the potential health risks associated with exposure to these agents. Epidemiological studies have reported associations between exposure to PFAS and a wide range of adverse health outcomes including thyroid disorders [6], kidney disease [7], liver dysfunction [8], endocrine disruption [9], immune perturbation [10], and impaired fetal development [11]. These associations are partially supported by *in vivo* and in vitro studies that suggested PFAS-induced thyroid dysfunction [12], nephrotoxicity [13], hepatic lipid dysregulation [14], endocrine effects [15], altered cytokine production [16], developmental toxicity [17], and carcinogenicity [18].

Furthermore, in recent years, exposure to PFAS has been linked to cardiovascular diseases, including increased cardiovascular events [19], promotion of atherosclerotic lesions and thrombosis [20], and elevated cardiovascular mortality [21], suggesting that PFAS serve as important risk factors for the onset and progression of cardiovascular diseases. Vascular endothelial cells, which line the luminal surface of blood vessels, regulate essential vascular functions such as coagulation, fibrinolysis, vascular permeability, inflammatory signaling, and angiogenesis [22]. Experimental studies have suggested that PFAS impair vascular endothelial angiogenesis, enhance inflammatory responses, induce cell death [23,24], and disrupt tight junctions, thereby increasing vascular permeability [25], suggesting that PFAS-induced endothelial dysfunction contributes to the development of cardiovascular disease.

Programmed cell death is the fundamental mechanism that eliminates damaged cells and maintains tissue homeostasis [26]. In addition to apoptosis, other regulated cell death pathways such as necroptosis and pyroptosis play crucial roles in pathological processes. Apoptosis proceeds in a caspase-dependent manner, with the extrinsic pathway activating Caspase-8 and the intrinsic pathway activating Caspase-9, both converging on Caspase-3/7 [27,28,29]. When Caspase-8 activity is impaired, necroptosis can proceed through the activation of the RIP1–RIP3 complex and subsequent MLKL-mediated membrane permeabilization [30,31]. Caspase-8 also suppresses necroptosis by cleaving RIP1 and RIP3. Pyroptosis is mediated by Caspase-1, which promotes the maturation of inflammatory cytokines and activates gasdermin D, leading to the formation of membrane pores and inflammatory cell death [32,33]. PFAS have been shown to activate multiple regulated cell death pathways depending on their chemical structure and the cell type to which they are exposed. PFOA induces apoptosis in human trophoblast cells through endoplasmic reticulum stress [34,35]. Perfluorodecanoic acid (PFDA) activates the RIPK3–MLKL pathway and triggers necroptosis of human granulosa cells [36]. PFOS activates AIM2 inflammasome and induces Caspase-1-dependent pyroptosis in human monocytic leukemia cells and mouse bone marrow-derived macrophages [37]. These findings indicate that the toxicity of PFAS is not restricted to a single canonical cell death pathway, but involves distinct modes of programmed cell death influenced by carbon chain length, functional groups, and cell-specific susceptibility.

Despite the growing evidence of PFAS-induced vascular endothelial toxicity, previous studies have only evaluated individual PFAS, limited cell types, and single toxicity endpoints. Systematic comparisons of structurally diverse PFAS under standardized experimental conditions remain scarce, and the molecular basis of PFAS-induced endothelial injury has not been fully elucidated. In this study, we comprehensively evaluated the cytotoxicity of 15 PFAS comprising perfluoroalkyl carboxylic acids and perfluoroalkyl sulfonates with varying carbon-chain lengths, together with representative alternative PFAS, in vascular endothelial cells under uniform conditions. By examining structure–activity relationships, cell-type specificity, and potential involvement of distinct programmed cell death pathways, we characterized the cytotoxic profiles and underlying cellular responses associated with exposure to PFAS in vascular endothelial cells. In the present study, BAECs were used as the principal endothelial model for morphological and mechanistic analyses, whereas additional human-, porcine-, and non-endothelial cell models were included to examine whether PFAS-associated changes observed in BAECs might also be seen across other cell systems under the same exposure conditions.

## 2. Materials and Methods

### 2.1. Materials

Bovine aortic endothelial cells (BAECs) and bovine aortic smooth muscle cells (BASMCs) were purchased from Cell Applications (San Diego, CA, USA). Human vascular endothelial EA.hy926 cells and bovine kidney epithelial (MDBK) cells were obtained from ATCC (Manassas, VA, USA). Human lung fibroblasts (IMR-90) and porcine kidney epithelial (LLC-PK1) cells were purchased from DS Pharma Biomedical (Osaka, Japan). Dulbecco’s modified Eagle’s medium (DMEM) and calcium- and magnesium-free phosphate-buffered saline (CMF-PBS) were obtained from Shimadzu Diagnostics (Tokyo, Japan). Fetal bovine serum (FBS) and tissue culture materials were purchased from Thermo Fisher Scientific (Waltham, MA, USA). The following PFAS were used in this study: perfluoropentanoic acid (PFPeA), perfluorohexanoic acid (PFHxA), perfluoroheptanoic acid (PFHpA), perfluorooctanoic acid (PFOA), perfluorononanoic acid (PFNA), perfluorodecanoic acid (PFDA), perfluorobutanesulfonate (PFBS) (Sigma-Aldrich, St. Louis, MO, USA); perfluoropentanesulfonate (PFPeS), perfluorohexanesulfonate (PFHxS), perfluoroheptanesulfonate (PFHpS), and perfluorooctanesulfonate (PFOS) (Toronto Research Chemicals, North York, ON, Canada); perfluorobutyric acid (PFBA), perfluorodecanesulfonate (PFDS), and 1H,1H,2H,2H-perfluorooctanesulfonate (6:2 FTS) (SynQuest Laboratories, Alachua, FL, USA); and undecafluoro-2-methyl-3-oxahexanoic acid (GenX) (Matrix Scientific, Columbia, SC, USA). Z-VAD-FMK was purchased from Peptide Institute (Osaka, Japan). Necrostatin-1 was purchased from Adipogen Life Sciences (San Diego, CA, USA). Polyvinylidene difluoride (PVDF) membranes (0.2 μm pore size) were obtained from Cytiva (Marlborough, MA, USA). The rabbit polyclonal anti-RIP3 antibody (SAB5700803) was purchased from Sigma-Aldrich (St. Louis, MO, USA). Rabbit polyclonal anti-caspase-1 antibody (22915-1-AP) was obtained from Proteintech (Rosemont, IL, USA) and rabbit polyclonal anti-caspase-8 antibody (NB100-56116) was obtained from Novus Biologicals (Centennial, CO, USA). Rabbit monoclonal anti-caspase-3 (#9665), rabbit monoclonal anti-phospho-RIP3 (#93654), and horseradish peroxidase-conjugated anti-rabbit IgG (#7074) antibodies were purchased from Cell Signaling Technology (Danvers, MA, USA). Rabbit polyclonal anti-MLKL (ab194699) and rabbit monoclonal anti-phospho-MLKL (ab187091) antibodies were purchased from Abcam (Cambridge, UK). Horseradish peroxidase-conjugated anti-glyceraldehyde-3-phosphate dehydrogenase (GAPDH) monoclonal antibody (015-25473) and bromophenol blue were purchased from FUJIFILM Wako Pure Chemical Industries (Osaka, Japan). ISOGEN II was purchased from Nippon Gene (Tokyo, Japan). Thunderbird SYBR qPCR Mix and ReverTra Ace qPCR RT Master Mix were purchased from TOYOBO (Osaka, Japan). May–Grünwald and Giemsa staining solutions, 3-(4,5-dimethyl-2-thiazolyl)-2,5-diphenyltetrazolium bromide (MTT), sodium dodecyl sulfate (SDS), BCA Protein Assay Kit, Chemi-Lumi One Super, and other reagents were purchased from Nacalai Tesque (Kyoto, Japan).

### 2.2. Cell Culture and Treatment

BAECs, BASMCs, as well as EA.hy926, MDBK, IMR-90, and LLC-PK1 cells were cultured in DMEM supplemented with 10% FBS at 37 °C in a humidified atmosphere containing 5% CO_2_. For exposure experiments using PFAS, cells were seeded at a density of 5000 cells/cm^2^ and cultured under the above conditions for 72 h. Confluence was confirmed by visual inspection before PFAS exposure. After confirming confluence, the cells were incubated in serum-free DMEM containing PFAS (0.1, 0.5, 1, 5, 10, 20, 50, 75, or 100 µM) for 24 h in 6-, 24-, or 96-well plates. For exposure experiments, cells were used after reaching confluence to evaluate PFAS responses in established monolayers, as is commonly done in vascular endothelial cell studies to model the vascular lining in vitro. Serum-free DMEM was used during PFAS exposure to minimize interactions between PFAS and serum components and to evaluate cellular responses under defined conditions.

### 2.3. Morphological Observation

The BAECs were cultured to confluence in 24-well plates. The cells were washed twice with DMEM and then incubated with DMEM containing PFAS (1, 10, or 100 µM) for 24 h. After exposure, the medium was removed, and the cells were washed with CMF-PBS. The cells were fixed with May–Grünwald staining solution for 3 min, and then stained with Giemsa solution diluted at a ratio of 1:20 with distilled water for 30 min. After washing with distilled water, the cells were air-dried overnight. Morphological changes were observed under an inverted microscope (DM IL LED; Leica Microsystems, Wetzlar, Germany) at 50× magnification.

### 2.4. MTT Assay

Cells were cultured to confluence in 96-well plates and washed twice with DMEM. The cells were then incubated with DMEM (100 µL) containing PFAS (0.1, 0.5, 1, 5, 10, 20, 50, 75, or 100 µM) for 24 h. After exposure, the culture medium was replaced with DMEM containing 10% FBS and MTT (0.25 mg/mL) and incubated for 4 h at 37 °C. The medium was removed and formazan crystals were dissolved in 100 µL dimethyl sulfoxide. Absorbance at 560 nm was measured using a microplate reader (GloMax Discover, Promega, Madison, WI, USA). Cell viability was expressed as a percentage relative to the untreated control cells. MTT linearity under the selected assay conditions had been confirmed in BAECs and EA.hy926 cells. The concentration producing a 50% reduction in cell viability (CC_50_) was determined from dose–response curves using nonlinear regression analysis with the Rodbard function implemented in ImageJ software (version 1.54s; National Institutes of Health, Bethesda, MD, USA).

### 2.5. Inhibitor Experiments

For inhibitor experiments, cells were pretreated with Z-VAD-FMK or necrostatin-1 (5, 10, 20, or 50 µM) for 1 h, followed by incubation with PFAS (100 µM) for 24 h.

### 2.6. Western Blotting

BAECs were cultured in 6-well plates in the presence or absence of PFAS (1, 10, 20, 50, or 100 µM) for 24 h. After incubation, the medium was removed, and the cells were lysed in 100 µL lysis buffer (50 mM Tris, 2% SDS, and 10% glycerol in pH 6.8), and then incubated at 95 °C for 10 min. Protein concentrations were determined using a BCA Protein Assay Kit (Nacalai Tesque). Glycerol, 2-mercaptoethanol, and bromophenol blue were added to the samples, which were then heated at 95 °C for 3 min. Whole-cell lysates were separated by SDS-polyacrylamide gel electrophoresis on 10% or 16% polyacrylamide gels and electrotransferred onto PVDF membranes at 2 mA/cm^2^ for 1 h. Membranes were blocked with 5% bovine serum albumin in 20 mM Tris-buffered saline containing 0.1% Tween-20 (TBST) for 1 h and incubated overnight at 4 °C with primary antibodies against Caspase-1, Caspase-8, RIP3, MLKL (1:2000 each), Caspase-3, phospho-RIP3, phospho-MLKL (1:1000 each), and GAPDH (1:10,000). After washing with TBST, the membranes were incubated with horseradish peroxidase-conjugated secondary antibodies (1:5000) for 1 h. The immunoreactive bands were detected by enhanced chemiluminescence using Chemi-Lumi One Super and visualized with an LAS4000 mini imaging system (FUJIFILM Wako Pure Chemical Industries). Band intensities were quantified using ImageJ software.

### 2.7. Real-Time Quantitative Reverse Transcription Polymerase Chain Reaction (Real Time RT-PCR)

BAECs were cultured in 6-well plates and incubated in the presence or absence of PFAS (1, 10, 20, 50, or 100 µM) for 24 h. Total RNA was extracted using ISOGEN II according to the manufacturer’s instructions. RNA concentration was determined using a NanoDrop spectrophotometer (Thermo Fisher Scientific). Complementary DNA (cDNA) was synthesized using the ReverTra Ace qPCR RT Master Mix. Real-time RT-PCR was performed using the Thunderbird SYBR qPCR Mix with 5 ng of cDNA on a StepOnePlus Real-Time PCR System (Thermo Fisher Scientific). The thermal cycling conditions were 95 °C for 20 s, followed by 40 cycles of 95 °C for 15 s and 60 °C for 45 s. Relative mRNA expression levels were calculated using the standard curve method and normalized to GAPDH expression. The bovine gene-specific primer sequences used were as follows: *RIP3*, 5′-CGACCTCCTCAGACTCCAGA-3′ (forward) and 5′-GTGTCACATCTCTCAGCCCC-3′ (reverse); *MLKL*, 5′-ACTTCCATCAGCCGACAAAC-3′ (forward) and 5′-CTCCCAGAGGACAATTCCAA-3′ (reverse); GAPDH, 5′-AACACCCTCAAGATTGTCAGCAA-3′ (forward) and 5′-ACAGTCTTCTGGGTGGCAGTGA-3′ (reverse).

### 2.8. Statistical Analysis

Data are expressed as means ± standard error (S.E.) from three or six independent experiments. Statistical significance was evaluated using Student’s *t*-test or one-way analysis of variance (ANOVA), followed by the Bonferroni/Dunn post hoc test for multiple comparisons. Differences were considered statistically significant at *p* < 0.05. All statistical analyses were performed using Statcel4 software (OMS Publishing, Tokyo, Japan).

## 3. Results

### 3.1. Effects of PFAS on Endothelial Morphology and Cell Viability

**Figure 1:**
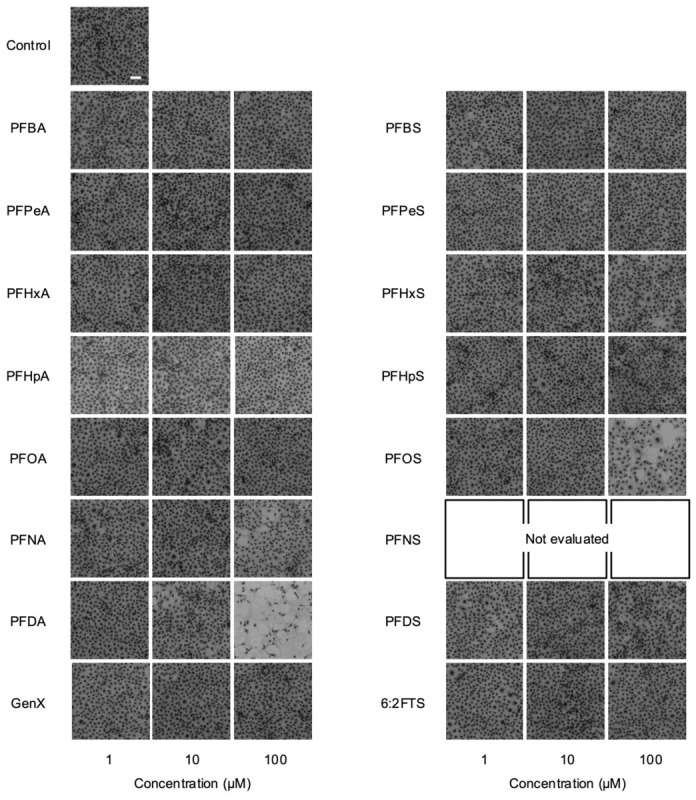
Morphological appearances of bovine aortic endothelial cells after exposure to PFAS. Confluent cultures of bovine aortic endothelial cells (BAECs) were incubated in the presence or absence of PFAS (1, 10, or 100 µM) for 24 h. The cell layer was then stained with Giemsa. Original magnification, ×50. Scale bar, 100 µm. PFNS was not evaluated.

The PFAS examined in the present study are listed in Table 1. The cytotoxicity of PFAS with carbon chain lengths ranging from C4 to C10 was examined in vascular endothelial cells (Figure 1). Among perfluoroalkyl carboxylic acids (PFCAs), compounds with shorter carbon chains (C4–C7), including PFBA, PFPeA, PFHxA, and PFHpA, did not cause detectable damage to the vascular endothelial cell layer at 100 µM (Figure 1, left panels). In contrast, longer-chain PFCAs, namely PFOA (C8), PFNA (C9), and PFDA (C10), markedly disrupted the endothelial cell layer. Among the perfluoroalkyl sulfonates (PFSAs), PFBS (C4), PFPeS (C5), PFHxS (C6), PFHpS (C7), and PFDS (C10) did not induce endothelial damage (Figure 1, right panels), whereas PFOS (C8) caused a clear disruption of the endothelial cell layer. Perfluorononane sulfonate (PFNS; C9) was not evaluated because of its unavailability. The alternative agent GenX and 6:2 fluorotelomer sulfonate (6:2 FTS) did not exhibit detectable endothelial cytotoxicity at 100 µM.

**Figure 2:**
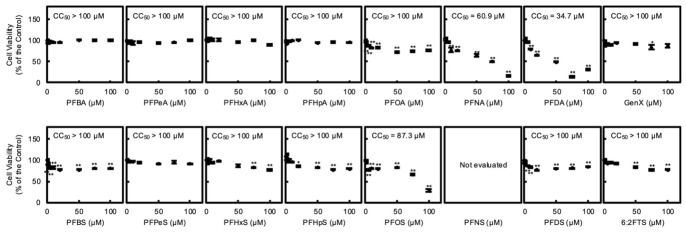
Cell viability of BAECs after exposure to PFAS. Confluent cultures of BAECs were incubated in the presence or absence of PFAS (0.1, 0.5, 1, 5, 10, 20, 50, 75, or 100 µM) for 24 h, and cell viability was measured using the MTT assay. The concentration producing a 50% reduction in cell viability (CC_50_) was determined using ImageJ software. Values are presented as means ± standard error (S.E.) of six independent samples. Statistical significance compared with the corresponding cells without PFAS: * *p* < 0.05, ** *p* < 0.01.

Next, PFAS-induced changes in endothelial cell viability were quantitatively evaluated using the MTT assay (Figure 2). Among PFCAs, PFBA, PFPeA, PFHxA, and PFHpA did not reduce endothelial cell viability up to 100 µM. In contrast, PFNA and PFDA significantly decreased cell viability, with CC_50_ values of 60.9 µM and 34.7 µM, respectively (Table 2). PFOA caused only a modest reduction in endothelial cell viability, without a clear concentration-dependent trend. Among the PFSAs, PFBS, PFHxS, and PFHpS slightly decreased endothelial cell viability, but did not show a clear concentration-dependent decrease. In contrast, PFOS markedly reduced endothelial cell viability with a CC_50_ value of 87.3 µM. PFPeS, GenX, and 6:2 FTS did not significantly affect endothelial cell viability.

### 3.2. Cell Type-Dependent Effects of PFAS

**Figure 3:**
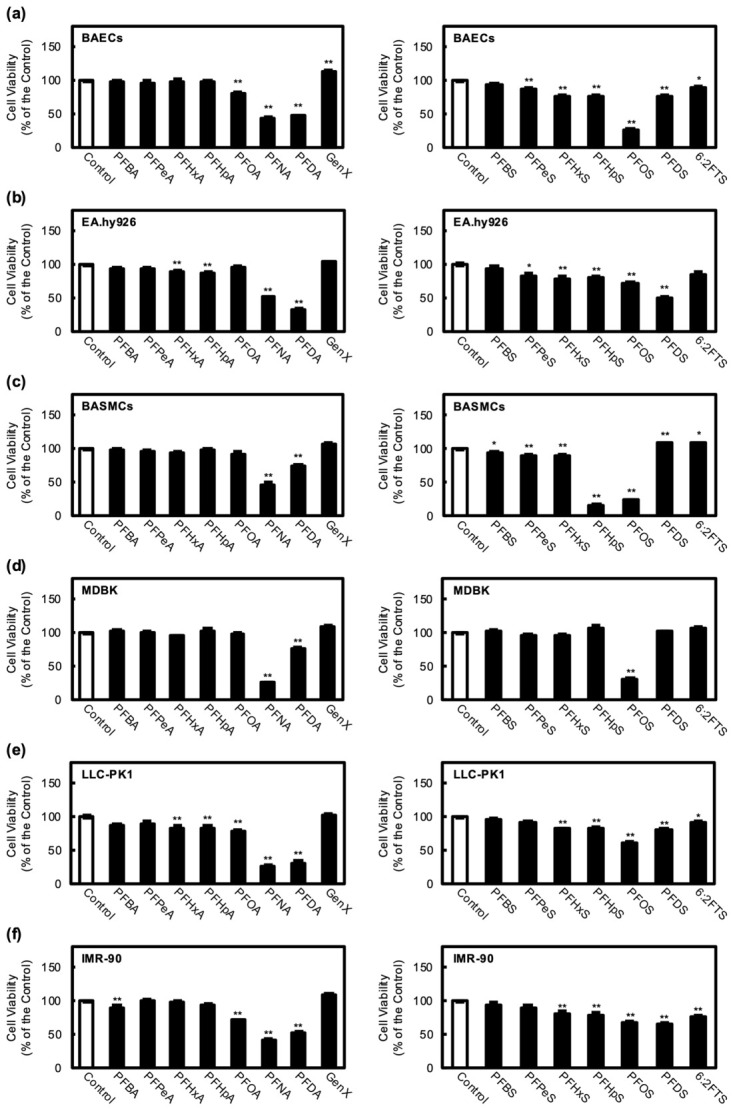
Effects of PFAS on cell viability in vascular endothelial cells, vascular smooth muscle cells, renal epithelial cells, and fibroblasts. Confluent cultures of (**a**) BAECs, (**b**) human vascular endothelial EA.hy926 cells, (**c**) bovine aortic smooth muscle cells (BASMCs), (**d**) bovine kidney epithelial cells (MDBK), (**e**) porcine kidney epithelial LLC-PK1 cells, and (**f**) human fetal fibroblastic IMR-90 cells were incubated in the presence or absence of PFAS (100 µM) for 24 h. Cell viability was then measured using the MTT assay. Values are presented as means ± S.E. of six independent samples. Statistical significance compared with the corresponding cells without PFAS, * *p* < 0.05, ** *p* < 0.01.

To evaluate cell type-dependent differences in PFAS responses, cell viability was assessed using the MTT assay in BAECs, BASMCs, and EA.hy926, MDBK, LLC-PK1, and IMR-90 cells (Figure 3). PFNA and PFDA, which caused endothelial damage in BAECs, also reduced cell viability in EA.hy926 cells, BASMCs, MDBK cells, LLC-PK1 cells, and IMR-90 cells (Figure 3, left panels). PFOA caused a relatively mild reduction in cell viability in BAECs, LLC-PK1 cells, and IMR-90 cells. PFOS reduced cell viability in most examined cell types. The reduction in cell viability induced by PFOS was greater in BAECs, BASMCs, and MDBK cells, whereas EA.hy926, LLC-PK1, and IMR-90 cells showed comparatively lower sensitivities. Although PFHpS did not cause marked endothelial damage in BAECs (Figure 1 and Figure 2), it reduced cell viability in several cell types including EA.hy926 cells, BASMCs, LLC-PK1 cells, and IMR-90 cells. PFHpS caused a greater reduction in cell viability than PFOS in BASMCs.

### 3.3. Effects of Apoptosis and Necroptosis Inhibitors on PFAS-Induced Endothelial Injury

**Figure 4:**
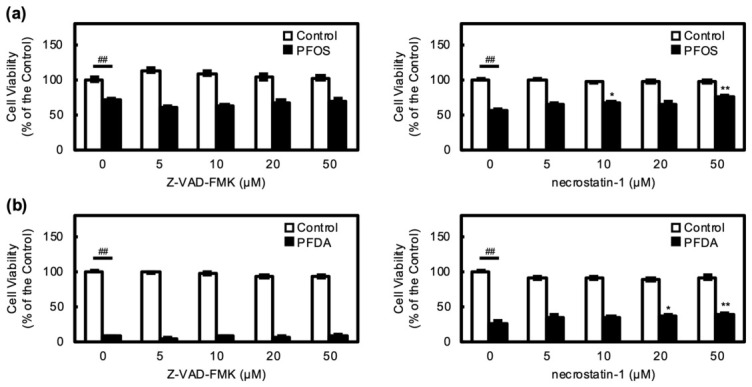
Cell death pathways involved in PFOS- and PFDA-induced injury in BAECs. Confluent cultures of BAECs were pretreated with the apoptosis inhibitor Z-VAD-FMK or the necroptosis inhibitor necrostatin-1 (5, 10, 20, or 50 µM) for 1 h, followed by incubation with or without (**a**) PFOS (100 µM) or (**b**) PFDA (100 µM) for 24 h. Cell viability was measured using the MTT assay. Values are presented as means ± S.E. of six independent samples. Statistical significance compared with the corresponding PFAS-treated cells without inhibitors, * *p* < 0.05, ** *p* < 0.01, and statistical significance compared with the corresponding cells without PFAS and inhibitors, ^##^ *p* < 0.01.

Since PFDA and PFOS were the most cytotoxic compounds among the PFCAs and PFSAs, respectively, in BAECs, the following experiments focused on these two compounds to compare their cell death signaling. The involvement of apoptosis and necroptosis in PFOS- and PFDA-induced reduction in cell viability in vascular endothelial cells was evaluated using the MTT assay (Figure 4). The quantitative summary of Figure 4 is provided in Table S1. The reduction in the viability of vascular endothelial cells after exposure to PFOS was not affected by pretreatment with the apoptosis inhibitor Z-VAD-FMK. In contrast, pretreatment with the necroptosis inhibitor necrostatin-1 partially restored cell viability from 56.5 ± 1.1% to 74.5 ± 2.8% of control (Figure 4a). Similar results were observed for PFDA, where Z-VAD-FMK had no effect, whereas necrostatin-1 partially restored cell viability from 25.7 ± 3.3% to 39.0 ± 2.5% of control (Figure 4b), suggesting that necroptosis is involved, in part, in PFOS- and PFDA-induced endothelial injury.

### 3.4. Activation of Cell Death Signaling Pathways in PFOS-Treated Endothelial Cells

**Figure 5:**
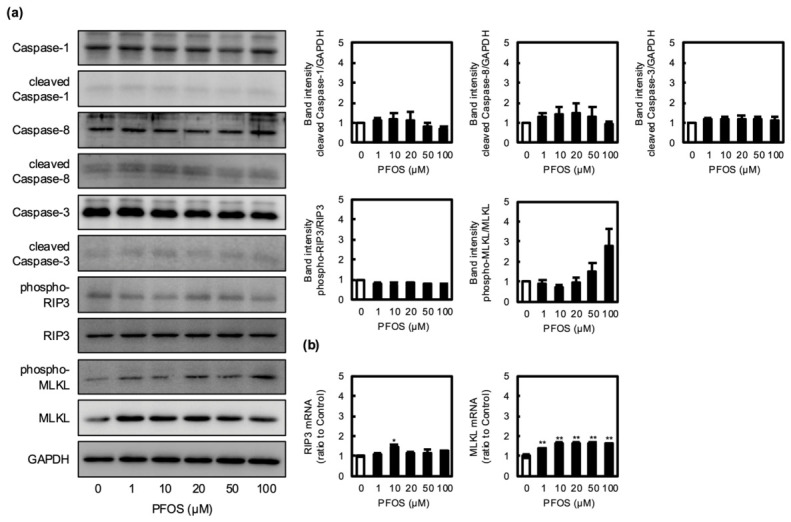
Activation of cell death signaling pathways in BAECs after exposure to PFOS. (**a**,**b**) Confluent cultures of BAECs were incubated in the presence or absence of PFOS (1, 10, 20, 50, or 100 µM) for 24 h. (**a**) Protein expression levels of Caspase-1, Caspase-3, Caspase-8, phosphorylated RIP3 (phospho-RIP3), RIP3, phosphorylated MLKL (phospho-MLKL), MLKL, and GAPDH were determined by Western blotting. GAPDH was used as a loading control. Phospho-RIP3 and phospho-MLKL were quantified as phospho-RIP3/RIP3 and phospho-MLKL/MLKL ratios, respectively. Values are presented as means ± S.E. of three independent samples. Statistical significance compared with the corresponding cells without PFOS, ** *p* < 0.01. (**b**) The expression levels of RIP3 and MLKL mRNAs were determined by real-time reverse transcription polymerase chain reaction (RT-PCR). Values are presented as means ± S.E. of three technical replicates. Statistical significance compared with the corresponding cells without PFOS, * *p* < 0.05, ** *p* < 0.01.

To characterize the mode of PFOS-induced endothelial cell death, the expression of cell death–related proteins was analyzed using Western blotting and real-time RT-PCR (Figure 5). The cleaved form of Caspase-1, a marker of pyroptosis, was not detected after PFOS treatment (Figure 5a). In addition, the protein levels of cleaved Caspase-8 and cleaved Caspase-3, markers of apoptosis, were not altered by PFOS treatment. Under the tested conditions, the selected pyroptosis- and apoptosis-related markers were not altered by PFOS treatment. The protein levels of phosphorylated RIP3 and total RIP3 remained unchanged, but both phosphorylated MLKL and total MLKL protein levels were increased by PFOS treatment. MLKL mRNA expression was also elevated (Figure 5b). These results suggest that PFOS-induced endothelial injury was associated with increased MLKL without detectable RIP3 activation under the tested conditions.

### 3.5. Activation of Cell Death Signaling Pathways in PFDA-Treated Endothelial Cells

**Figure 6:**
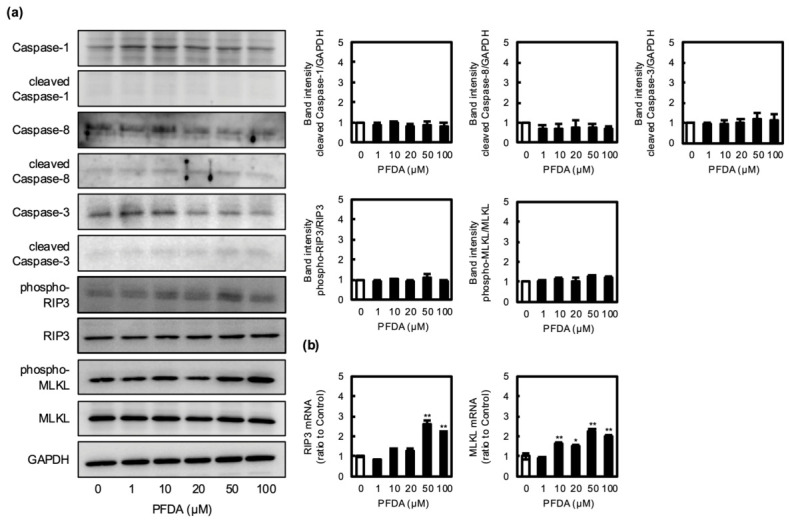
Activation of cell death signaling pathways in BAECs after exposure to PFDA. (**a**,**b**) Confluent cultures of BAECs were incubated in the presence or absence of PFDA (1, 10, 20, 50, or 100 µM) for 24 h. (**a**) Protein expression levels of Caspase-1, Caspase-3, Caspase-8, phospho-RIP3, RIP3, phospho-MLKL, MLKL, and GAPDH were determined by Western blotting. GAPDH was used as a loading control. Phospho-RIP3 and phospho-MLKL were quantified as phospho-RIP3/RIP3 and phospho-MLKL/MLKL ratios, respectively. Values are presented as means ± S.E. of three independent samples. Statistical significance compared with the corresponding cells without PFDA. (**b**) The expression levels of RIP3 and MLKL mRNAs were determined by real-time RT-PCR. Values are presented as means ± S.E. of three technical replicates. Statistical significance compared with the corresponding cells without PFDA, * *p* < 0.05, ** *p* < 0.01.

The mechanism of PFDA-induced endothelial cell death was also examined (Figure 6). PFDA treatment did not alter the protein levels of cleaved Caspase-1, cleaved Caspase-8, or cleaved Caspase-3 (Figure 6a), indicating that the selected pyroptosis- and apoptosis-related markers were not altered under the tested conditions. In contrast, PFDA treatment increased the protein levels of phosphorylated RIP3, total RIP3, phosphorylated MLKL, and total MLKL. Additionally, RIP3 and MLKL mRNA expression levels were elevated (Figure 6b). These findings suggest that PFDA-induced endothelial injury was associated with increases in phosphorylated RIP3 and phosphorylated MLKL under the tested conditions.

## 4. Discussion

The present study had several major findings. First, PFNA, PFDA, and PFOS reduced cell viability in vascular endothelial cells; among the PFCAs, endothelial cytotoxicity increased with increasing carbon chain length. Second, PFDA reduced cell viability broadly across the cell types examined, whereas PFOS showed a greater reduction in cell viability in the bovine-derived cell types examined than in the human- or porcine-derived cell types examined. Third, PFDA and PFOS did not alter the selected apoptosis- and pyroptosis-related markers under the tested conditions. Fourth, PFOS was associated with increased MLKL protein and mRNA expression. Fifth, PFDA was associated with increases in phosphorylated RIP3 and phosphorylated MLKL. These findings suggest that both the molecular structure of PFAS and the characteristics of target cells contribute to PFAS-induced vascular endothelial injury.

The toxicity of chemical substances is strongly influenced by their molecular structure, and differences in the carbon-chain length and functional groups of PFAS are known to affect both cellular toxicity and intracellular behavior [38]. The increased hydrophobicity associated with longer carbon chains enhances interactions with cellular membranes and may increase intracellular accumulation [39,40]. In the present study, PFCAs showed increased endothelial cytotoxicity with increasing carbon chain length, suggesting that enhanced membrane interactions and an altered affinity for intracellular molecules may have contributed to the observed toxicity. In contrast, among the PFSAs examined, PFOS exhibited marked cytotoxicity, whereas the longer-chain PFDS did not cause clear endothelial injury. These findings indicate that the cytotoxicity of PFSAs cannot be explained solely by carbon chain length. Alternative PFAS, such as GenX and 6:2 FTS, which possess shorter or branched structures, have been reported to exhibit lower bioaccumulation than legacy PFAS [41], and the absence of marked endothelial cytotoxicity observed in the present study is consistent with these characteristics.

Toxic responses to chemical substances can vary depending on cell type and type of compound, which is an important factor when extrapolating in vitro findings to humans. PFSAs, including PFOS, are known substrates of organic anion transporters [42,43], and differences in the expression or activity of these transporters may influence their cellular uptake [11,44]. In addition, PFAS are known to bind serum albumin, and both legacy and alternative PFAS have been reported to exhibit higher binding affinities for human serum albumin than for bovine serum albumin [45], suggesting that the affinity of PFAS for proteins differs across species. In the present study, however, PFAS exposure was performed under serum-free conditions, making it unlikely that differences attributable to serum albumin itself directly accounted for the observed responses. Rather, species-dependent differences in the recognition of PFAS by other proteins, including secreted proteins and membrane proteins such as receptors and transporters, may also have influenced cellular responses. Interestingly, PFHpS induced cytotoxicity in multiple cell types, and exhibited stronger toxicity than PFOS in bovine aortic smooth muscle cells. This finding suggests that PFAS toxicity may depend not only on the molecular structure but also on the characteristics of the target vascular cell type.

Differences in the mode of cell death can substantially influence inflammatory responses and tissue injury [26]. Necroptosis is a programmed form of necrotic cell death executed through the RIP3–MLKL signaling pathway and is characterized by membrane rupture and release of inflammatory mediators, which can amplify tissue inflammation [30,31]. In vascular endothelial cells, inflammatory cell death is closely associated with vascular dysfunction, including increased vascular permeability and activation of coagulation pathways. Recent studies have reported that environmental pollutants, such as heavy metals and polycyclic aromatic hydrocarbons, can induce necroptosis as a mechanism of toxicity [46,47]. Moreover, MLKL-dependent endothelial necroptosis has been implicated in systemic inflammation and thrombotic responses [48]. In the present study, PFDA was associated with increases in phosphorylated RIP3 and phosphorylated MLKL, whereas PFOS increased MLKL expression without detectable RIP3 activation. These findings suggest that PFDA and PFOS were associated with distinct necroptosis-related signaling patterns under the tested conditions. By contrast, PFOA did not show clear changes in the cell death-related markers examined, suggesting that such signaling changes were not uniformly observed across PFAS under the present experimental conditions. MLKL has been reported to be activated independently of RIP3 or through phosphorylation by alternative kinases [49,50], and the strong membrane affinity and intracellular accumulation of PFOS may contribute to activation of such noncanonical pathways. However, necrostatin-1 only partially restored cell viability, indicating that necroptosis alone may not fully explain PFAS-induced endothelial injury and suggesting the involvement of additional cell death mechanisms.

The present study has several limitations. First, the concentrations of the PFAS used in this study, particularly those associated with marked endothelial injury, were higher than those typically detected in environmental and human exposure settings and were not intended to quantitatively reproduce PFAS concentrations in the human aorta. However, relatively high concentrations are often used in in vitro mechanistic studies to identify potential toxicity-related mechanisms of chemical substances. Second, this study primarily evaluated acute responses, and the effects of long-term exposure to lower concentrations of PFAS were not examined. Therefore, some of the molecular changes observed in the present study are considered to reflect secondary responses associated with advanced cellular injury, in addition to the direct effects of PFAS. PFOS and long-chain PFCAs have been reported to impair mitochondrial function [51,52]. Since the MTT assay reflects mitochondrial activity, its readout is influenced by PFAS and does not necessarily fully represent cellular injury. Because it served as the principal quantitative endpoint in the present study, the current data do not fully distinguish among reduced cell number, altered mitochondrial activity, differences in monolayer confluence, and overt cell death. In addition, reduced MTT signals were observed even for PFAS and at concentrations that did not produce overt endothelial injury in morphological observations, suggesting that metabolic activity declines before overt cell death becomes apparent. Thus, PFAS may affect cellular function even at concentrations that do not induce overt cell death. A complementary orthogonal assay for cell death was not included in the present study. MTT linearity under the selected conditions had been confirmed in BAECs and EA.hy926 cells, but not across all additional cell models and plate formats. Confluence was confirmed by visual inspection before PFAS exposure, and stricter comparison among biologically distinct cell models would require more rigorous validation. Third, because the present study was conducted using cultured cells, the experimental conditions did not fully reflect an in vivo environment, including pharmacokinetics and inter-organ interactions. In addition, mechanistic analyses were restricted to BAECs; PFAS uptake or free-concentration estimates were not examined, pathway-positive controls were not included, and endothelial functional endpoints were not evaluated. Nevertheless, the present study provides a systematic comparison of structurally diverse PFAS under the same experimental protocol and reveals the characteristic features of PFAS-induced vascular endothelial injury. In particular, the reduction in cell viability induced by PFCAs increased with carbon chain length, whereas PFSAs exhibited response patterns that could not be explained solely by this aspect. Moreover, PFOS showed cell type-dependent sensitivity, and both PFOS and PFDA were associated with necroptosis-related signaling patterns. These findings provide important insights into the molecular mechanisms underlying PFAS-induced vascular endothelial toxicity.

## 5. Conclusions

The present study showed that PFAS-induced endothelial injury differs according to molecular structure and cell type. PFNA, PFDA, and PFOS reduced endothelial cell viability, and PFOS and PFDA were associated with distinct necroptosis-related signaling patterns in BAECs. These findings provide a basis for understanding the structure-dependent endothelial toxicity of PFAS, although further studies are needed to clarify their significance under more physiologically relevant conditions.

## Figures and Tables

**Table 1:** Perfluoroalkyl and polyfluoroalkyl substances examined in this study.

Compound	Abbreviation	Carbon Number	Functional Group	CAS Number
Perfluoroalkyl carboxylic acids (PFCAs)				
Perfluorobutyric acid	PFBA	C4	Carboxylic acid	375-22-4
Perfluoropentanoic acid	PFPeA	C5	Carboxylic acid	2706-90-3
Perfluorohexanoic acid	PFHxA	C6	Carboxylic acid	307-24-4
Perfluoroheptanoic acid	PFHpA	C7	Carboxylic acid	375-85-9
Perfluorooctanoic acid	PFOA	C8	Carboxylic acid	335-67-1
Perfluorononanoic acid	PFNA	C9	Carboxylic acid	375-95-1
Perfluorodecanoic acid	PFDA	C10	Carboxylic acid	335-76-2
Perfluoroalkyl sulfonates (PFSAs)				
Perfluorobutanesulfonate	PFBS	C4	Sulfonate	375-73-5
Perfluoropentanesulfonate	PFPeS	C5	Sulfonate	3872-25-1
Perfluorohexanesulfonate	PFHxS	C6	Sulfonate	355-46-4
Perfluoroheptanesulfonate	PFHpS	C7	Sulfonate	375-92-8
Perfluorooctanesulfonate	PFOS	C8	Sulfonate	2795-39-3
Perfluorodecanesulfonate	PFDS	C10	Sulfonate	335-77-3
Alternative PFAS				
Undecafluoro-2-methyl-3-oxahexanoic acid	GenX	—	Ether carboxylic acid	13252-13-6
1H,1H,2H,2H-Perfluorooctanesulfonate	6:2 FTS	6:2	Fluorotelomer sulfonate	27619-94-9

**Table 2:** The concentration producing a 50% reduction in cell viability (CC_50_) and 95% confidence intervals (CI) for PFAS-induced cytotoxicity.

Compound	Carbon Number	CC50 (µM)	95% CI (µM)
PFCAs			
PFBA	C4	>100	Not estimable
PFPeA	C5	>100	Not estimable
PFHxA	C6	>100	Not estimable
PFHpA	C7	>100	Not estimable
PFOA	C8	>100	Not estimable
PFNA	C9	60.9	53.4 to 68.4
PFDA	C10	34.7	28.4 to 41.0
PFSAs			
PFBS	C4	>100	Not estimable
PFPeS	C5	>100	Not estimable
PFHxS	C6	>100	Not estimable
PFHpS	C7	>100	Not estimable
PFOS	C8	87.3	82.3 to 91.2
PFDS	C10	>100	Not estimable
Alternative PFAS			
6:2 FTS	6:2	>100	Not estimable
GenX	—	>100	Not estimable

CC_50_ values were estimated using a four-parameter logistic/Rodbard nonlinear regression model. Values are based on *n* = 6 wells per concentration. For compounds that did not reduce cell viability to 50% within the tested concentration range, CC_50_ was recorded as >100 µM, and the 95% confidence interval was not estimable.

## Data Availability

The data presented in this study are available upon request from the corresponding author.

## Supplementary Materials

The following supporting information can be downloaded at https://www.mdpi.com/article/10.3390/toxics14060510/s1: Table S1: Quantitative summary of Figure 4.
