# Supplementary material for: Comparative Analysis of Structurally Diverse PFAS-Induced Injury in Vascular Endothelial Cells and Characterization of Necroptosis-Related Cell Death Signaling
Source: Toxics. 2026 Jun 11;14(6):510. doi: 10.3390/toxics14060510 (PMC13306966; doi:10.3390/toxics14060510)
Supplement: Supplementary file 1 [file toxics-14-00510-s001.zip › toxics-4323662-supplementary.pdf]

Supplementary Table S1. Quantitative summary of Figure 4.

Panel	PFAS	Inhibitor	Exposure	Inhibitor conc. (μM)	n	Cell viability (% of control)	Effect size vs PFAS alone	95% CI	Exact p value
A (left)	PFOS	Z-VAD-FMK	-	0	6	100.0 ± 3.8	—	—	—
A (left)	PFOS	Z-VAD-FMK	-	5	6	113.1 ± 2.8	—	—	—
A (left)	PFOS	Z-VAD-FMK	-	10	6	108.1 ± 3.4	—	—	—
A (left)	PFOS	Z-VAD-FMK	-	20	6	104.0 ± 4.4	—	—	—
A (left)	PFOS	Z-VAD-FMK	-	50	6	101.5 ± 3.1	—	—	—
A (left)	PFOS	Z-VAD-FMK	+	0	6	69.8 ± 3.3	—	—	—
A (left)	PFOS	Z-VAD-FMK	+	5	6	60.4 ± 2.6	-9.4	-18.8 to -0.0	0.0491
A (left)	PFOS	Z-VAD-FMK	+	10	6	61.7 ± 2.0	-8.1	-16.7 to 0.4	0.0605
A (left)	PFOS	Z-VAD-FMK	+	20	6	67.5 ± 3.9	-2.3	-13.7 to 9.1	0.6626
A (left)	PFOS	Z-VAD-FMK	+	50	6	68.3 ± 4.9	-1.5	-14.6 to 11.7	0.8076
A (right)	PFOS	Necrostatin-1	-	0	6	100.0 ± 0.9	—	—	—
A (right)	PFOS	Necrostatin-1	-	5	6	100.5 ± 1.0	—	—	—
A (right)	PFOS	Necrostatin-1	-	10	6	96.5 ± 0.4	—	—	—
A (right)	PFOS	Necrostatin-1	-	20	6	97.5 ± 1.8	—	—	—
A (right)	PFOS	Necrostatin-1	-	50	6	97.1 ± 1.8	—	—	—
A (right)	PFOS	Necrostatin-1	+	0	6	56.5 ± 1.1	—	—	—
A (right)	PFOS	Necrostatin-1	+	5	6	64.1 ± 2.0	+7.6	2.3 to 13.0	0.0114
A (right)	PFOS	Necrostatin-1	+	10	6	66.6 ± 1.6	+10.1	5.6 to 14.6	0.000665
A (right)	PFOS	Necrostatin-1	+	20	6	64.3 ± 4.3	+7.8	-3.2 to 18.8	0.131
A (right)	PFOS	Necrostatin-1	+	50	6	74.5 ± 2.8	+18.0	10.9 to 25.1	0.000660
B (left)	PFDA	Z-VAD-FMK	-	0	6	100.0 ± 4.9	—	—	—
B (left)	PFDA	Z-VAD-FMK	-	5	6	61.0 ± 3.8	—	—	—
B (left)	PFDA	Z-VAD-FMK	-	10	6	60.2 ± 3.2	—	—	—
B (left)	PFDA	Z-VAD-FMK	-	20	6	58.6 ± 4.5	—	—	—
B (left)	PFDA	Z-VAD-FMK	-	50	6	53.3 ± 3.3	—	—	—
B (left)	PFDA	Z-VAD-FMK	+	0	6	7.2 ± 1.7	—	—	—
B (left)	PFDA	Z-VAD-FMK	+	5	6	4.4 ± 0.9	-2.7	-7.1 to 1.6	0.1940
B (left)	PFDA	Z-VAD-FMK	+	10	6	7.2 ± 1.9	+0.0	-5.7 to 5.7	0.9933
B (left)	PFDA	Z-VAD-FMK	+	20	6	5.9 ± 1.6	-1.3	-6.5 to 3.9	0.5947
B (left)	PFDA	Z-VAD-FMK	+	50	6	8.3 ± 1.6	+1.1	-4.1 to 6.4	0.6381
B (right)	PFDA	Necrostatin-1	-	0	6	100.0 ± 1.0	—	—	—
B (right)	PFDA	Necrostatin-1	-	5	6	90.5 ± 2.0	—	—	—
B (right)	PFDA	Necrostatin-1	-	10	6	90.7 ± 1.8	—	—	—
B (right)	PFDA	Necrostatin-1	-	20	6	88.2 ± 2.5	—	—	—
B (right)	PFDA	Necrostatin-1	-	50	6	91.4 ± 2.4	—	—	—
B (right)	PFDA	Necrostatin-1	+	0	6	25.7 ± 3.3	—	—	—
B (right)	PFDA	Necrostatin-1	+	5	6	34.7 ± 2.6	+9.0	-0.4 to 18.4	0.0587
B (right)	PFDA	Necrostatin-1	+	10	6	34.1 ± 2.3	+8.4	-0.6 to 17.4	0.0633
B (right)	PFDA	Necrostatin-1	+	20	6	37.0 ± 1.1	+11.4	3.6 to 19.1	0.00845
B (right)	PFDA	Necrostatin-1	+	50	6	39.0 ± 2.5	+13.4	4.1 to 22.6	0.00906

Cell viability values are shown as mean ± S.E. Effect size was calculated as the difference in normalized cell viability relative to the corresponding PFAS-alone group.

Exact p values and 95% confidence intervals are shown for comparisons with the corresponding PFAS-alone group.
